# Effects of Amorphous Calcium Phosphate Administration on Dental Sensitivity during In-Office and At-Home Interventions

**DOI:** 10.3390/dj6040052

**Published:** 2018-10-01

**Authors:** Giacomo Oldoini, Antonino Bruno, Anna Maria Genovesi, Luca Parisi

**Affiliations:** 1 Stomatologic Institute of Tuscany, Foundation of Clinic, Research and Post Graduate Program, 55041 Camaiore (Lucca), Italy; giacomo.oldoini88@gmail.com (G.O.); formazione@istitutostomatologicotoscano.it (A.M.G.); 2 Laboratory of Vascular Biology and Angiogenesis, Scientific and Technological Pole, IRCCS MultiMedica, 20138 Milan, Italy; 3 Department of Biomedical, Surgical and Dental Sciences, School of Dentistry, University of Milan, 20122 Milan, Italy; luca.parisi@unimi.it

**Keywords:** dental sensitivity, tooth bleaching, amorphous calcium phosphate, in-office procedures, at-home procedures

## Abstract

Background. Tooth bleaching is the most frequently employed whitening procedure in clinics. The major side effect of tooth bleaching is dental sensitivity during and after the treatment. Here, we evaluated whether the administration of amorphous calcium phosphate (ACP), during in-office and at-home procedures may impact on dental sensitivity. Methods. Eighty patients, responding to the study requirements were enrolled according to the following criteria. Group 1 (*n* = 40), received in-office, 10% ACP prior to 30% professional hydrogen peroxide application. The whitening procedure continued at home using 10% carbamide peroxide with 15% ACP for 15 days. Group 2 (*n* = 40) received only 30% hydrogen peroxide application and continued the whitening procedures at home, using 10% carbamide hydroxide, without ACP- Casein phosphopeptides (CPP), for 15 days. Dental sensitivity was recorded with a visual analogue scale (VAS) at baseline, immediately after, and at 15 days after treatment in the two groups. Results. We observed that patients receiving ACP in the bleaching mixture experienced decreased dental sensitivity (* *p* ≤ 0.05), as detected by VAS scale analysis immediately following the procedures. Patients receiving ACP-CPP during at-home procedures showed a statistically significant (*** *p* ≤ 0.0001) reduction of dental sensitivity. Conclusions. We demonstrated that ACP-CPP administration, while exerting the same whitening effects as in control subjects receiving potassium fluoride (PF), had an impact on the reduction of dental sensitivity, improving patient compliance.

## 1. Introduction

Oral diseases are among the most prevalent chronic diseases worldwide, representing a burden to health-care services. Treatments of dental diseases are expensive, accounting for between 5% and 10% of total health-care expenditures in industrialized countries [1]. Demineralization is a process naturally occurring within the oral cavity, as a consequence of a drop in pH that, if it persists, results in caries induction and tooth loss [2]. Dentinal hypersensitivity is a common oral condition characterized by pain resulting from dentine exposure to chemical, thermal, tactile or osmotic factors [3].

Tooth bleaching is a safe and conservative treatment modality to improve the aesthetic appearance of discolored teeth. Optimal results, along with the low costs for this procedure, have resulted in an increasing use of tooth bleaching in clinical dentistry [4,5]. In association with this, a plethora of new bleaching agents have been tested and introduced to the market. These are characterized by different percentages in the composition of their active principles, such as carbamide peroxide and carbamide, diverse routes of exposition to those agents and different disposal techniques.

Despite being a totally safe procedure, one of the major undesirable effects of bleaching is dentin sensitivity, which may occur during and after treatment, thus representing a degree of biological damage affecting the dentin–pulp complex [6].

Within the large number of techniques described in the literature concerning the external bleaching of vital teeth, all are based on the direct use of hydrogen peroxide (H_2_O_2_) or its precursor, carbamide peroxide [7]. Hydrogen peroxide (H_2_O_2_) is employed as a whitening agent at concentrations ranging from 25% to 35% [8,9]. Nevertheless, high concentrations of H_2_O_2_, especially in patients with elevated enamel permeability, or those where prolonged use of bleaching agents has been reported, resulted in increased dental sensitivity.

Dentinal hypersensitivity represents a very common effect following different whitening treatments. The association of agents able to reduce dental sensitivity during bleaching procedures still represents a major issue. The gold standard for professional products is not combined with products for the reduction of post-treatment sensitivity, lacking dedicated protocols and entrusting the management of the problem to the use of generic fluorine [10].

Treatment with 35% H_2_O_2_ has been associated with alteration of the nervous dentinal activities, both when treatment has been performed with, or without, mineralizing calcium [11]. There are additional risks that have been reported from in vitro studies, including tooth erosion, tooth mineral degradation, increased susceptibility to demineralization, and pulpal damage [12].

Given these dental sensitivity issues, diverse manufacturers are investing their efforts into developing bleaching gels with lower concentrations of H_2_O_2_. This aims to minimize the side effects related to the development of peroxide radicals [6,13], when using a bleaching agent with elevated peroxide content.

Along with dental sensitivity, another concern associated with bleaching procedures is represented by the stability of the teeth color over time. Diverse strategies have been employed to overcome both of these issues, including professional (referred as in-office) and in-house bleaching procedures.

In-house treatments aim at reducing adversities related to dentinal hypersensitivity. Jorgensen et al. demonstrated that in-house treatments resulted in a decreased severe sensitivity. The population analyzed showed that 50% of the subjects had low sensitivity, 10% moderate sensitivity and only 4% severe sensitivity [14]. However, following two weeks of treatment, the hypersensitivity was completely abrogated in all the subjects receiving the treatment [14].

Several studies showed that in 60–90% of cases, patients reported increased sensitivity when receiving professional treatment. Therefore, to prevent and/or reduce the problems of sensitivity, different approaches were employed, such as combination with 2-hydroxyethyl-glutaraldehyde (G2H) [15]. A study comparing the effects of G2H against a placebo during in-office treatment, showed a significant reduction of dentinal sensitivity, without considering the overall aesthetic effect on teeth color [16].

Casein phosphopeptides (CPP) represents another agent used for reducing dentinal sensitivity. CPP can selectively deliver Ca^2+^, (PO_4_)^3−^ ions and fluorine within the tooth enamel [17]. CPP can be associated with carriers as for amorphous calcium phosphate (ACP). ACP has been largely used by dentists, given its versatility and that it has been positively associated with caries inhibition [18], mineralizing activities [19], the inhibition of caries-induced tooth enamel demineralization [18], and increased white spot [20,21]. 

An in vitro study investigated the ability of casein to inhibit tooth enamel demineralization as related to fluoride [20]. One hundred and twenty (*n* = 120) blocks of tooth enamel were exposed to three different solutions containing casein, fluoride or variable pH. The ACP-treated group showed significantly decreased (*p* = 0.05) demineralization, as compared to those treated with fluoride, and the other two groups. Therefore, the CPP-ACP combination was more effective in preserving the intact surface of the whitened tooth, as confirmed by scanning electron microscopy (SEM) analysis [10]. 

A recent meta-analysis summarized major studies on the efficacy of fluorides and ACP-CPP vs fluorides as monotherapy. Interestingly very few studies have reported on the impact of ACP-CPP administration to dental sensitivity, suggesting the urgency of this poorly explored topic [22].

The quantity and quality of clinical trial evidence is insufficient to support the long-term effectiveness of casein derivatives, specifically ACP-CPP, in preventing caries in vivo and treating dentin hypersensitivity or dry mouth. We aim to investigate whether the use of ACP, combined with CPP can result in increased comfort in patients, by reducing dental sensitivity through the treatment.

## 2. Materials and Methods

### 2.1. Patient Selection

Patients recruited within the study were enrolled after obtaining informed consent in an institutional ethics committee-approved study. The study was performed under a clinically approved protocol (n°421, 16-03-2016), by the Ethics Committee—Milan-Area B, Ospedale Maggiore, Fondazione IRCCS Ca Granda, Ospedale Maggiore Policlinico Milano.

Investigations were carried out following the principles of the Declaration of Helsinki of 1975. Eighty patients, responding to the study requirements were enrolled according to the following criteria.

*Inclusion:*

Age ≥ 18
Overall good healthy condition
With all the dentary settings
Smokers and non-smokers

*Exclusion:*

Dental hypersensitivity
Known periodontal problems
Subjected to bleaching within 1 year
Pregnancy
Bleeding On Probing (BOP) > 5%, Plaque Index (PI) > 18%

### 2.2. Study Groups

Patients were divided into two groups as follows: Group 1: 40 patients received, in-office, 15% ACP-CPP prior to professional 30% hydrogen peroxide application. Group 1 continued the whitening procedures at home using 10% carbamide-hydroxide with the 15% ACP-CPP for 15 days.

Group 2: 40 patients received, in office, only 30% hydrogen peroxide. Group 2 continued the whitening procedures at home, using 10% carbamide peroxide without ACP-CPP for 15 days. Two independent professionals enrolled the patients according to the indications from the literature. 

### 2.3. Procedures

Two weeks prior to bleaching, all the patients received professional hygiene procedures using an ultrasonic scaler (Mectron Combi Touch, Mectron s.p.a, Genova, Italy) and spherical powder based on calcium carbonate (Prophylaxis Powder Smooth–Mectron s.p.a, Genova, Italy). The use of this powder seems to be adapted for this scope to polish the teeth before a dental bleaching procedure. During the professional hygiene procedures, several indicators were registered, such as the plaque and bleeding index. The at-home hygiene procedures included the use of interdental brushes (Gum Soft Picks, regular to large size), sonic toothbrush (Philips Sonicare, Seattle, WA, USA), toothpaste nano idrossiapatite (Biorepair-Cosweell, Bologna, Italy), in association with ACP relief (Philiphs, Seattle, WA, USA) application (once per day). Patients were monitored at 2 and 4 weeks following the first application. The VAS (visual analogue scale) system was used to determine the dental sensitivity event [13,23] and every patient directly provided the VAS value. VAS analysis was performed before treatment (T0), immediately following the treatment (T1), 15 days (T2) and 30 days (T3) following treatment. The variation in the dental color tone was evaluated at T0 and T3.

The indications for the at-home procedures were as follows:

- Sonicare Philips toothbrush
- Biorepair toothpaste containing nano-hydroxyapatite
- Use of an interdental toothbrush

### 2.4. Statistical Analysis

Data were analyzed using Graphpad-Prism7. *t*-test student for statistical analysis. Statistical significance was considered at * *p* ≤ 0.05. 

## 3. Results

### 3.1. Patient Characteristics

Forty patients received ACP-CPP supplementation and were compared with forty controls. Patients had an average age of 36 ± 12.28 (mean ± sd), of which 44 were males and 36 females. 

### 3.2. ACP-CPP Administration during Bleaching Procedures Reduces Dental Sensitivity Both in-Office and at Home

**Figure 1:**
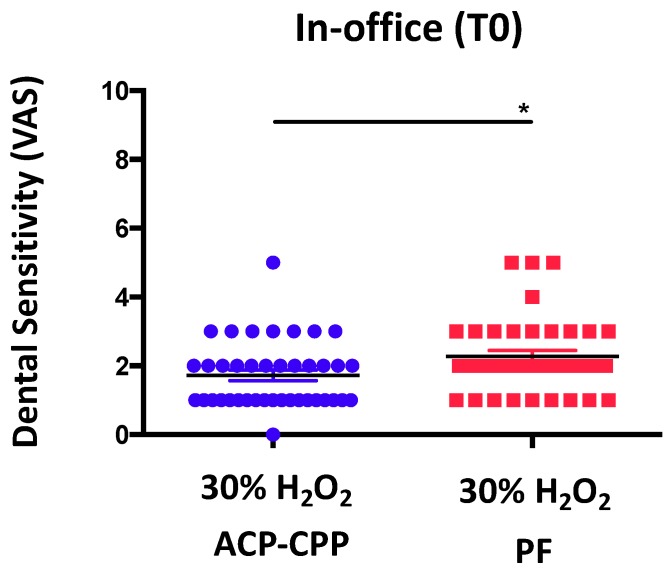
**Effects of ACP-CPP combination on dental sensitivity, following in-office oral hygiene procedures.** ACP-CPP treatment significantly reduced dental sensitivity, as compared to the control group (PF), immediately after treatment (T1). Results are showed as mean ± SEM, * *p* ≤ 0.05.

**Figure 2:**
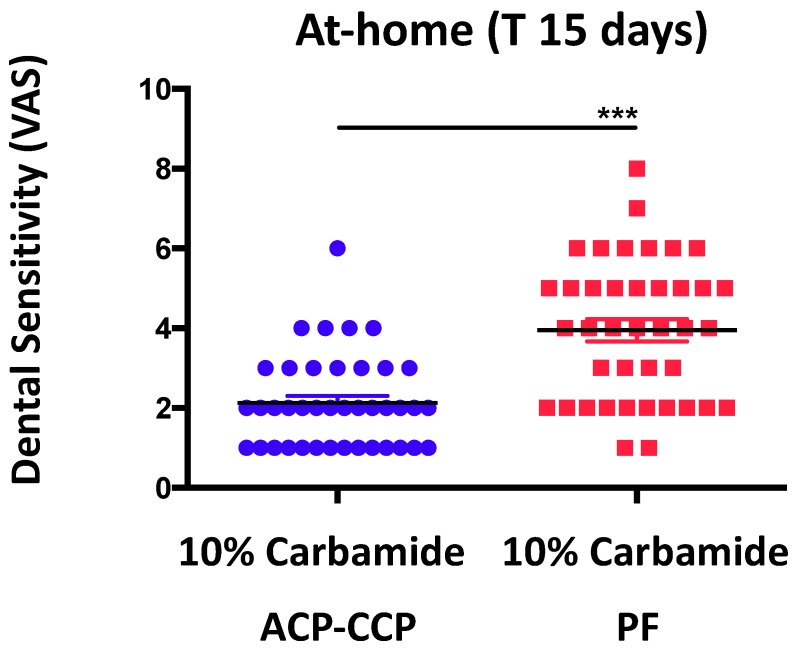
**Effects of ACP-CPP combination on dental sensitivity, following at-home oral hygiene procedures.** ACP-CPP treatment significantly reduced dental sensitivity as compared to control (PF) group, during the following 15 (T15) days. Results are showed as mean ± SEM, *** *p* ≤ 0.0001.

We observed that patients receiving ACP-CPP in the bleaching mixture experienced decreased dental sensitivity (* *p* ≤ 0.05), as detected by VAS scale analysis, immediately following the procedures (Figure 1). We also found that patients receiving ACP-CPP during at-home procedures showed a highly statistically significant (*** *p* ≤ 0.0001) reduction of dental sensitivity (Figure 2).

**Figure 3:**
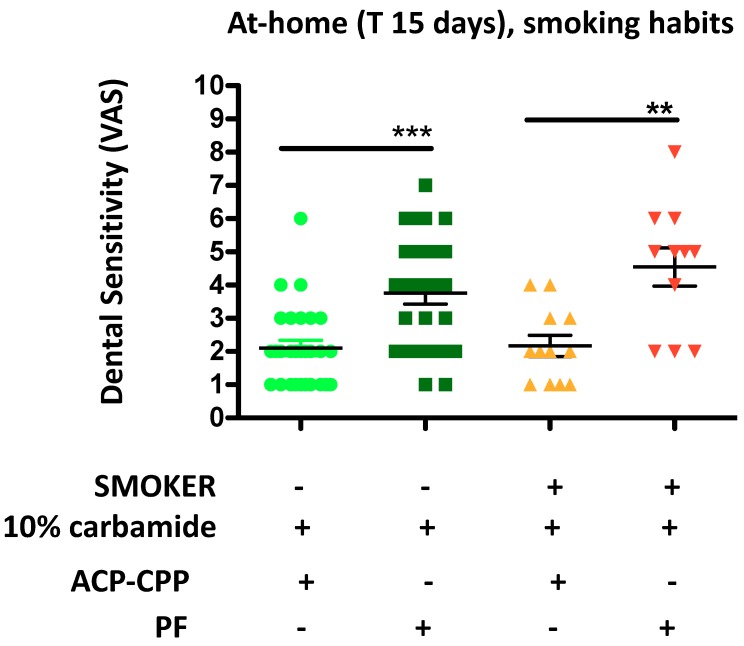
Effects of ACP-CPP administration on dental sensitivity, following at-home oral hygiene procedures as related to smoking habits. ACP-CPP treatment resulted in decreased dental sensitivity, as compared with the control (PF) group, both in non-smoker and smoker patients. Results are showed as mean ± SEM, ** *p* ≤ 0.01; *** *p* ≤ 0.0001.

Finally, we determined that ACP-CPP can reduce dental sensitivity both in non-smoking (*** *p* ≤ 0.0001) and smoking (*** *p* ≤ 0.0001) patients (Figure 3).

## 4. Discussion

Tooth bleaching (whitening) is one of the most common and economic methods to treat the discoloration of teeth [24]. Dental aesthetics, especially tooth color, is of great importance for the majority of people; and teeth discoloration can negatively influence the quality of life, especially from a social point of view [25]. The increasing demand for tooth bleaching has driven many manufacturers and researchers to develop whitening products to be used either in the dental office or at home [24]. However, as for any dental procedure, bleaching involves risks that include increased tooth sensitivity and mild gingival irritation [6,23,24,26,27]. The development of these side effects is directly related to the concentration of the hydrogen peroxide bleach component, duration of the treatment, and the non-bleach composition of the product used [28]. Therefore, strategies aimed at limiting bleaching-associated dental sensitivity during both in-office and at-home procedures are urgently needed.

A systematic review and meta-analysis to evaluate the risk and intensity of tooth sensitivity during in-office and at-home bleaching in adult patients revealed that no differences can be detected, either regarding the risk/intensity of tooth sensitivity or the effectiveness of the bleaching treatment [29]. This comparison, however, does not take into consideration variations in the protocols (daily usage time, number of bleaching sessions, and product concentration) of the bleaching techniques in the studies included [29]. It is now clear that the use of diverse agents combined with whitening compounds might impact on dental sensitivity and can shift towards a significant difference in reducing this main concern related to dental whitening. 

In line with this relevant issue, we investigated whether ACP-CPP administration might impact on dental sensitivity, a major complication occurring during bleaching procedures. Dental sensitivity was measured using the VAS scale, which is routinely employed in clinics. We first evaluated the effects of ACP immediately following in-office bleaching procedures.

We showed that ACP supplementation during both in-office and at-home procedures significantly decreased the dental sensitivity. Patients receiving ACP-CPP experienced reduced dental sensitivity both in office, immediately following bleaching, and at home. Discomfort during bleaching procedures is a major issue in the specific field [30], thus the combination of intervention procedures joining together rapid aesthetic effects with reduced pain for the patients are urgently needed. In this view, aimed at limiting the discomfort for the patients that must frequently attend in-office bleaching, we determined whether ACP-CPP administration during at-home procedures might limit dental sensitivity. We found that the administration of ACP-CPP during both in-office and at-home procedures reduced the dental sensitivity and minimized the use of anti-inflammatory agents, which is otherwise necessary in cases of high dental sensitivity, improving patient compliance.

## 5. Conclusions

Our work demonstrated that the use of ACP-CPP during at-home bleaching procedures, by improving the patient compliance, reduced both patient discomfort during long and frequent in-office treatments/visits, as well as the costs, whilst making aesthetic treatment more easily available.
